# Effects of L-Ornithine-L-Aspartate on Angiogenesis and Perfusion in Subacute Hind Limb Ischemia: Preliminary Study

**DOI:** 10.3390/biomedicines12081787

**Published:** 2024-08-06

**Authors:** Sanghoon Jung, Ye Jin Park, Jiwon Jeon, Kyuseok Kim

**Affiliations:** 1 Department of Radiology, CHA University School of Medicine, Pocheon 13488, Gyeonggi-do, Republic of Korea; dugguijsh@gmail.com; 2 Department of Emergency Medicine, CHA University School of Medicine, Pocheon 13488, Gyeonggi-do, Republic of Korea; yejin6577@naver.com (Y.J.P.); tw7682@nate.com (J.J.)

**Keywords:** L-ornithine-L-aspartate, peripheral arterial disease, angiogenesis

## Abstract

The current treatment options for peripheral arterial disease (PAD) are limited due to a lack of significant high-level evidence to inform clinical decisions and unfavorable outcomes in terms of cost-effectiveness and amputation rates. In order to suggest the use of the commercially available L-Ornithine-L-Aspartate (LOLA) for treating PAD, we induced hind limb ischemia (HLI) by unilaterally ligating the femoral artery in a rat model. The rats were randomly divided into three groups, with seven rats assigned to each group: group 1 (control), group 2 (sorbitol), and group 3 (LOLA). Intraperitoneal injections were administered five times on post-operative days (PODs) 3, 5, 7, 10, and 12. Perfusion imaging was conducted on PODs 7 and 14 and compared to pre-operative perfusion imaging. Immunohistochemistry staining and Western blotting were performed after the final perfusion imaging. Group 3 showed a significant increase in perfusion, high CD31-positive capillary lumen density, and substantial overexpression of VEGF in the ischemic limb during the subacute phase of HLI. In conclusion, this study provides the first documented evidence of angiogenesis and perfusion recovery in the subacute phase of the HLI model following the administration of LOLA. With LOLA readily available on the commercial market, the implementation of LOLA treatment for PAD in humans can be expedited compared to other therapies still in the developmental stage.

## 1. Introduction

As global life expectancy increases, the number of elderly people affected by type II diabetes, obesity, and hypertension—established risk factors for atherosclerosis—has also increased. This has led to a rise in the incidence of peripheral arterial disease (PAD), caused by the progression of atherosclerotic plaque, affecting over 230 million adults worldwide [1,2]. Critical limb ischemia (CLI), the advanced stage of PAD, carries significant risks of amputation, stroke, myocardial infarction, and death, resulting in a lower quality of life [3,4].

In the United States alone, about 60,000 major amputations are performed each year, with a global decrease attributed to the increased use of endovascular procedures [5,6]. Endovascular intervention is considered the standard care for patients with peripheral artery disease (PAD), especially for high-risk individuals who are not eligible for open revascularization [7,8]. However, despite the rise in endovascular interventions, the number of minor amputations has increased while major amputations have decreased [9]. The outcomes of surgical or interventional revascularization are not optimal, as repeat interventions and major amputations are still necessary for up to one-third of patients with critical limb ischemia (CLI) treated by revascularization, leaving a significant portion of the population ineligible for these treatments [4,10]. Cilostazol, the only widely approved medication for intermittent claudication, has limited effectiveness and is not recommended for individuals with heart failure, leading to discontinuation by up to half of users due to dizziness or palpitations [11,12,13]. Despite advances in medical therapy, including antiplatelet agents and statins, and improvements in managing comorbidities to enhance limb salvage rates and reduce mortality, the overall survival rate of CLI patients remains lower than that of individuals with other serious conditions like heart failure and certain cancers, averaging around 3.5 years [14,15,16,17]. Considering the limitations of current CLI treatments, therapeutic neovascularization has emerged as a promising option for addressing ischemia, but its clinical application is slow and challenging [18,19].

Nitric oxide (NO) is a well-known regulator of endothelial function, playing a crucial role in endothelial vasorelaxation [20]. NO also upregulates vascular endothelial growth factor (VEGF), a key stimulatory factor for angiogenesis [21,22,23]. Endothelial nitric oxide synthase (eNOS) contributes to collateral arterial adaptation and blood flow recovery in a mouse hind limb ischemia (HLI) model [24]. However, the complex regulation of eNOS expression and activity, involving numerous interrelated mechanisms, currently lacks a clinically applicable treatment [25].

L-Ornithine-L-Aspartate (LOLA), an existing crystalline salt, is currently employed in the clinical management of hepatic encephalopathy [26,27]. L-ornithine can enter the mitochondria and be converted to L-citrulline, while L-aspartate facilitates the conversion of L-citrulline to L-arginine—the sole physiologically significant substrate for NO synthesis [28,29,30,31,32]. There are some reports on the role of L-arginine in angiogenesis in patients with chronically hypoxic lungs and its use as a concomitant treatment for surgical angiogenesis in severe diffuse coronary arterial disease patients [33,34]. However, the long-term administration of L-arginine is not efficient in patients with PAD, even though short-term L-arginine supplementation in patients with CLI induces NO-dependent peripheral vasodilation [35,36]. Although there is no current documentation of the administration of LOLA for CLI treatment, more efficient angiogenesis is expected with LOLA compared to direct administration of L-arginine due to its physiologic production of L-arginine and subsequent NO. Because LOLA is already commercialized with proven safety, there is a high possibility of rapid clinical translation if the efficacy is proven in preclinical studies. Accordingly, this rat model study of HLI is designed to evaluate the efficacy of LOLA as a treatment option for CLI, with the aim of swift translation to human application.

## 2. Materials and Methods

### 2.1. Animals

This study was approved by the Institutional Animal Care and Use Committee of the authors’ institute (IACUC-230034) in accordance with the National Institutes of Health Guidelines. This study was carried out in compliance with the ARRIVE guidelines. Male Sprague Dawley rats (Seong-Nam, Republic of Korea) weighing 280–320 g (8 weeks old) were used. The rats were housed in a controlled environment (room temperature 20–24 °C, humidity 40–60%) with access to standard food and water ad libitum during the experiment.

### 2.2. Hind Limb Ischemia

Anesthesia was administered using 5% isoflurane in O_2_. After anesthesia, analgesia, sedation, and muscle relaxation were performed using intramuscular injection of tiletamine and zolazepam (Zoletil, Virbac, Carros, France) mixed with xylazine (Rompum, Elanco, Indianapolis, IN, USA) (1:1). The concentration of each was 0.7 mg/kg. Then, rats underwent unilateral femoral artery ligation distally to the origin of the deep branch in the right leg. An oblique incision was made with Metzenbaum scissors along the inguinal area. The femoral nerve, artery, and vein were isolated following the removal of the fat pad. The tissue surrounding these structures was dissected using sterile cotton swabs and Graefe forceps. A ligature (4-0 Vicryl) was then tied around the right femoral artery near the inguinal ligament [1,37].

### 2.3. Protocol

Just before the operations, perfusion imaging was performed. Following the operations, immediate post-op perfusion imaging was performed; post-operative day 0. The rats were then divided into three groups by stratified randomization based on body weight, which was measured by a research assistant. We assigned 7 rats per group: group 1 (control group; injection of normal saline), group 2 (sorbitol group; injection of sorbitol), and group 3 (LOLA treatment group). Normal saline (1.7 mL/kg) in group 1, a mixture of distilled water, sorbitol, and normal saline (20:2:30, 1.7 mL/kg) in group 2, and LOLA (333 mg/kg) (Hepa-Merz infusion, Hanwha Pharma, Chuncheon, Republic of Korea) in group 3 were injected intraperitoneally five times on PODs 3, 5, 7, 10 and 12. Since Herpa-Merz infusion consists of LOLA, sorbitol, and distilled water, an equal amount of sorbitol and distilled water with normal saline instead of LOLA were administrated to group 2. For the control group, an equal amount of normal saline was assigned to group 1. Perfusion imaging was scanned on PODs 7 and 14. Immunohistochemical analysis and Western blotting of gastrocnemius muscles in ischemic limbs were performed after the final perfusion imaging.

### 2.4. Perfusion Imaging

The anesthesia method employed during surgery was used for each perfusion imaging session. Rats were subjected to imaging for approximately 3 min using a Laser Doppler imager (Moor LDI, Axminster, UK). Images were analyzed with Moor LDI Imaging Review. Regions of interest (ROIs) were drawn around feet and ankles, and perfusion ratios comparing post-operative ischemic limbs with pre-operative limbs were calculated.

### 2.5. Immunohistochemical Analysis 

The rats were euthanized, and their tissues were fixed with 4% paraformaldehyde 14 days after surgery. The muscles were removed, cut into blocks, embedded in paraffin, sectioned at 5 µm using a microtome, and then mounted on glass slides. The sections were incubated for 10 min with Proteinase K enzyme (S3020, Agilent DAKO, Santa Clara, CA, USA) at room temperature as a condition for antibody expression. Blocking was performed with 5% bovine serum albumin for 30 min. Subsequently, the sections were incubated for 1 h with antibodies against VEGF-A165 (1:100, MA1-16629, Thermo Scientific, Waltham, MA, USA) and CD31 (1:100, MA1-80069, Thermo Scientific, Waltham, MA, USA) at room temperature. The sections were then washed three times for 5 min each with Tris-Buffered Saline (TBS) buffer. Following this, the sections were incubated for 20 min at room temperature with a secondary antibody (Envi-112 envision + system-HRP Labeled Polymer—Anti-mouse, K4001, Agilent DAKO, Santa Clara, CA, USA). Subsequent to the incubation, the sections were washed three times for 5 min each with TBS buffer. Digital images of five microscopic fields from four different sections of each animal were captured and viewed using a Zeiss Slide scanner (ZENblue3.1_ZENblack_3-OSR-lite, Carl Zeiss Microscopy, LLC, White Plains, NY, USA; magnification, ×400). Capillary density was determined as the number of CD31-positive cells per square millimeter [38] and confirmed after a consensus review by two medical doctors for each case.

### 2.6. Western Blotting 

Frozen muscle samples were homogenized in RIPA Lysis and Extraction Buffer (Thermo Fisher Scientific, Waltham, MA, USA) with 1% Halt™ Protease Inhibitor Cocktail (Thermo Fisher Scientific, Waltham, MA, USA) and centrifuged at 13,000× *g* for 15 min at 4 °C. Protein concentrations were determined using a BCA assay kit (cat. no. 23250; Pierce; Thermo Fisher Scientific, Waltham, MA, USA), according to the manufacturer’s instructions. Equal amounts of protein were loaded onto 12% Tris-glycine SDS-Polyacrylamide gels and separated by SDS-PAGE (Bio-Rad, Hercules, CA, USA). Following electrophoresis, the gels were transferred to polyvinylidene difluoride (PVDF) membranes (GenDEPOT, Baker, TX, USA). The membranes were blocked for 1 h at room temperature in TBS (Tris-Buffered Saline) with Tween-20 buffer (Biosolution, Suwon, Republic of Korea) containing 5% bovine serum Albumin (BSA) (bioWORLD, Dublin, OH, USA). Membranes were incubated overnight at 4 °C with primary antibodies against VEGF-A_165_ (MA1-16629; Thermo Fisher Scientific, Waltham, MA, USA) and Glyceraldehyde 3-phosphate dehydrogenase (GAPDH) diluted at a ratio of 1:1000 in 5% BSA-TBS-T. The membranes were washed for 5 min, 5 times with TBS-T buffer, incubated for 1 h at room temperature with Goat Anti-mouse lgG antibody (HRP, GeneTex, Irvine, CA, USA) diluted at a ratio of 1:10,000 in 5% BSA-TBS-T, and washed for 5 min, 5 times with TBS-T buffer. Bands were detected using Clarity™ Western ECL Substrate (Bio-Rad, Hercules, CA, USA). VEGF-A_165_ is typically expressed as a 46 kDa homodimer of 23 kDa subunits [39]. Relative expression of 46 kDa homodimer was normalized to endogenous control GAPDH using ImageJ software version 4.1 (National Institutes of Health, Bethesda, MD, USA).

### 2.7. Statistical Analysis

Data were presented as the means ± standard deviation. One-way ANOVA test, Fisher’s least significant difference test, and an independent sample *t*-test were used for statistical analysis. A *p*-value < 0.05 was considered to indicate a statistically significant difference. The data were analyzed using SPSS software (version 29.0, SPSS, Inc., Chicago, IL, USA).

## 3. Results

**Figure 1:**
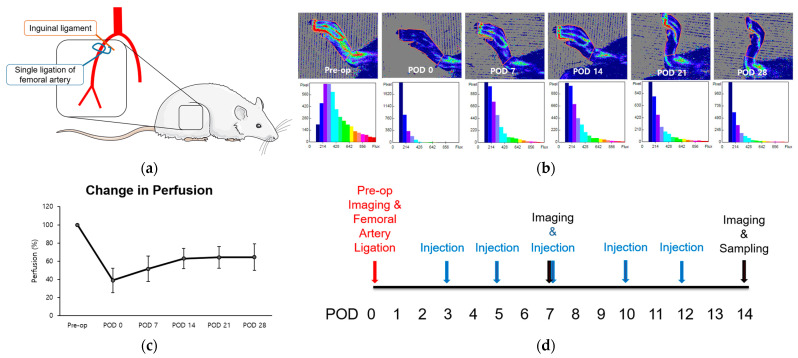
Change in perfusion after single ligation of proximal femoral artery in HLI model. (**a**) Schematics of the surgery. Rats underwent unilateral right femoral artery ligation as close as possible to the inguinal ligament (**b**). Laser Doppler perfusion imaging. Regions of interest (ROI) were drawn around feet and ankles, and perfusion ratios were calculated. (**c**) A point in linear plot of experiments representing percentage of perfusion recovery (*n* = 2). (**d**) Schematics of the protocol. After pre-operative perfusion imaging and surgery, substances corresponding to each group were injected intraperitoneally five times within two weeks of surgery. Perfusion imaging was scanned at PODs 7 and 14, followed by sampling of gastrocnemius muscles of ischemic limbs. HLI: hind limb ischemia; pre-op: pre-operative; POD: post-operative day.

The femoral arteries of two 8-week-old rats were unilaterally ligated to induce hind limb ischemia (HLI) and stimulate angiogenesis, particularly in the gastrocnemius muscle (Figure 1a) [1]. Immediate post-operative perfusion showed an approximate 40% reduction compared to normal pre-operative levels. Subsequently, perfusion increased over the 14-day period, reaching a plateau between weeks three and four at approximately 60% recovery (Figure 1a–c). The duration leading up to this plateau was defined as the perfusion recovery period. Using this model, we examined the alterations in perfusion and microenvironment to assess the impact of LOLA on angiogenesis during the subacute phase of HLI. LOLA was administered five times, starting from POD 3 and continuing for two weeks after surgery (Figure 1d).

### 3.1. Perfusion Changes

**Figure 2:**
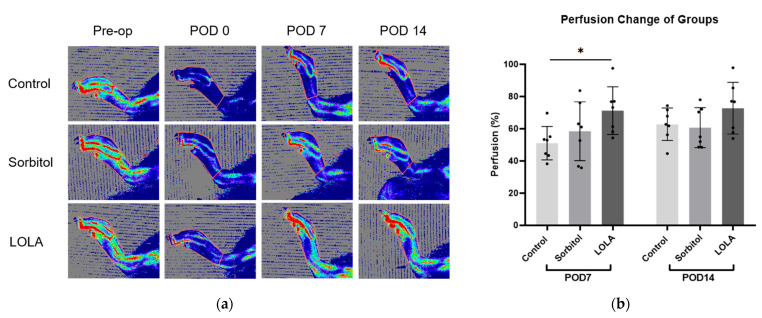
Perfusion recovery after administration of LOLA. (**a**) Laser Doppler perfusion imaging of three groups. Regions of interest (ROI) were drawn around feet and ankles, and perfusion ratios were calculated. Red represents the highest flow, and blue represents the lowest. (**b**) Bar plots of experiments representing percentage of perfusion recovery in three groups (group 1, blue; group 2, red; group 3, green). Error bar indicates standard error of mean. * indicates significant difference at *p* < 0.05. Group 1 (control; *n* = 7); group 2 (sorbitol; *n* = 7); group 3 (LOLA; *n* = 7); LOLA: L-Ornithine-L-Aspartate; POD: post-operative day.

On POD seven, the group administered LOLA (group 3) exhibited a significantly higher perfusion recovery rate compared to the control (group 1) and sorbitol-administered group (group 2) (51.05 ± 10.31 vs. 58.45 ± 18.24 vs. 71.22 ± 14.79; *p* < 0.05). Although not statistically significant, there was a tendency towards increased perfusion recovery at POD 14 in group 3 compared to the other groups (group 1 vs. group 2 vs. group 3: 62.77 ± 10.10 vs. 60.67 ± 12.45 vs. 72.76 ± 16.09; *p* = 0.211) (Figure 2).

### 3.2. Histological Evaluation for Capillary Density

**Figure 3:**
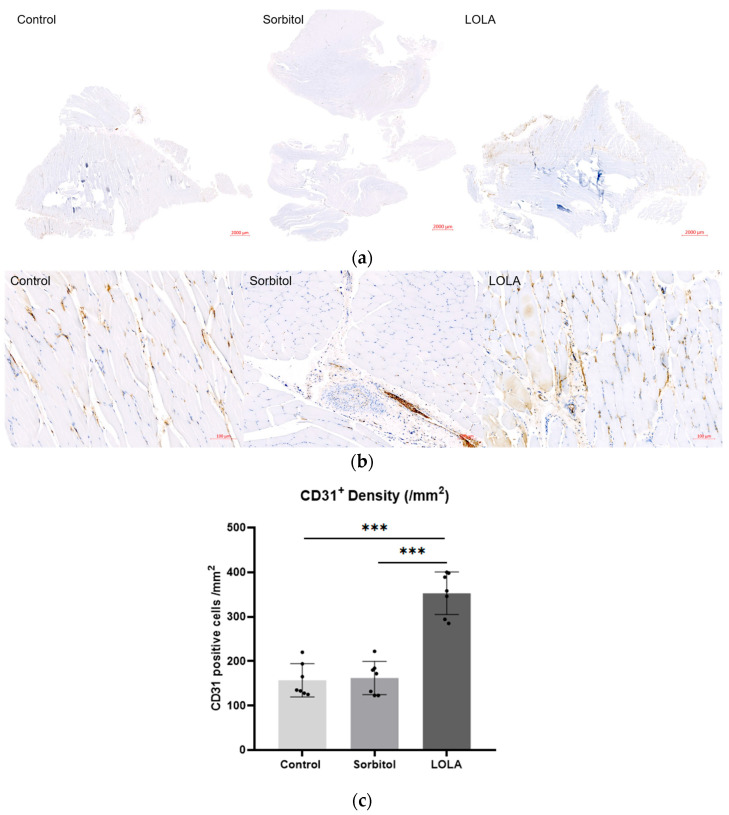
Angiogenesis after administration of LOLA. (**a**) Protein expression of CD 31 was detected by immunohistochemistry in gastrocnemius muscles of HLI rats. The scale bars indicate 2000 µm. (**b**) High magnification of (**a**). The scale bars indicate 100 µm. (**c**) CD31 positive cell density. Error bar indicates standard error of mean. *** indicate significant difference at *p* < 0.001. Group 1 (control; *n* = 7); group 2 (sorbitol; *n* = 7); group 3 (LOLA; *n* = 7); LOLA: L-Ornithine-L-Aspartate.

For a more in-depth examination of perfusion differences among the groups, muscle samples on POD 14 underwent immunohistochemistry targeting the endothelial cell marker CD31 to quantify vessel lumen density. The CD31-positive lumen density, indicative of capillary neovascularization, was significantly higher in rats treated with LOLA (group 3) compared to the other groups (157.14 ± 37.25 vs. 162.29 ± 37.53 vs. 352.86 ± 47.80; *p* < 0.001) (Figure 3).

### 3.3. Expression of VEGF

**Figure 4:**
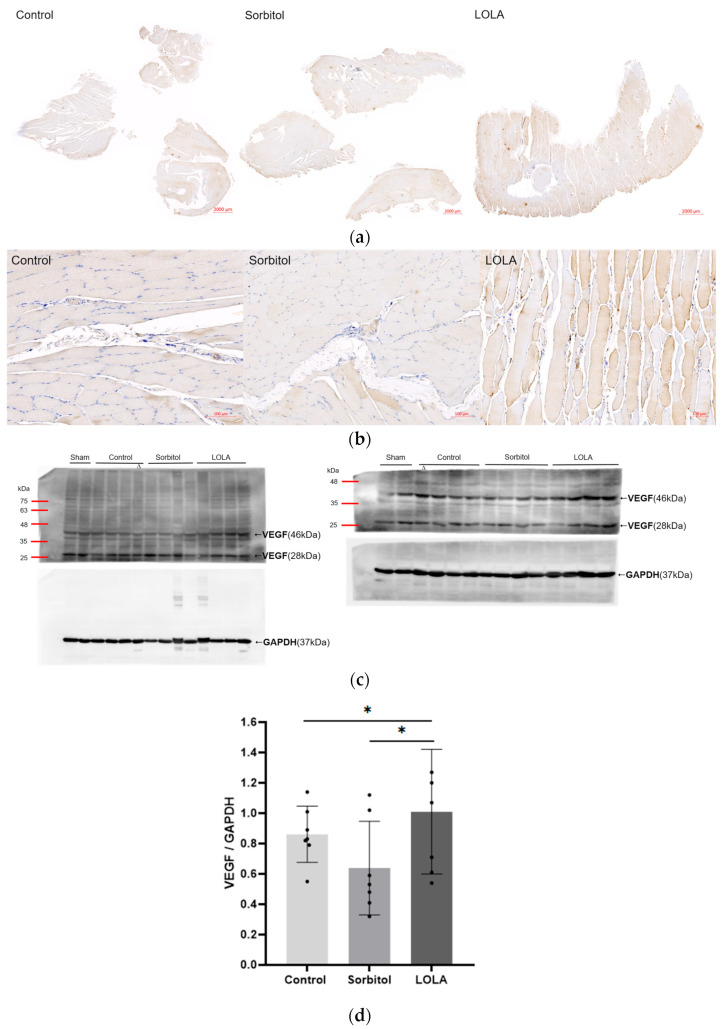
Angiogenesis after administration of LOLA. (**a**) Protein expression of VEGF was detected by immunohistochemistry in gastrocnemius muscles of HLI rats. The scale bars indicate 2000 µm. (**b**) High magnification of (**a**). The scale bars indicate 100 µm. (**c**) Western blotting of VEGF. Relative expression of a VEGF homodimer (46 kDa) of 23 kDa subunits was normalized to endogenous control GAPDH. Western blotting was incidentally performed on samples obtained from one rat of the control group (∆), and expression level was estimated from the initial result. Although Western blotting and some other analyses were conducted in a sham, the results are not included in this report. (**d**) VEGF expression level. Error bar indicates standard error of mean. * indicates significant difference at *p* < 0.05, respectively. Group 1 (control; *n* = 7); group 2 (sorbitol; *n* = 7); group 3 (LOLA; *n* = 7); GAPDH: Glyceraldehyde 3-phosphate dehydrogenase; LOLA: L-Ornithine-L-Aspartate; VEGF: vascular endothelial growth factor.

The levels of VEGF expression in the ischemic limb, as determined by Western blotting on POD 14, were notably higher in rats treated with LOLA (group 3) compared to the other groups (group 1 vs. group 2 vs. group 3: 0.86 ± 0.17 vs. 0.64 ± 0.28 vs. 1.01 ± 0.38; *p* < 0.05). Compared to the other groups, group 3’s immunohistochemical staining for VEGF was also enhanced (Figure 4).

## 4. Discussion

Our study using a rat model with LOLA administration demonstrated a significant increase in perfusion in the ischemic limb, high CD31-positive capillary lumen density, and substantial overexpression of VEGF during the subacute phase of HLI.

The pharmacokinetic and pharmacodynamic properties of LOLA are well-established in hepatic encephalopathy and it is already commercialized [26,27,40]. Our interest in the angiogenetic effect of L-arginine in patients with chronically hypoxic lung and asthma, along with endothelial modulation in patients with severe diffuse coronary arterial disease, prompted our investigation [33,34,41]. LOLA increases L-arginine via the conversion of L-ornithine, which is crucial for nitric oxide (NO) synthesis [28,29,30,31,32]. NO plays a vital role in angiogenesis by upregulating the vascular generation of VEGF and coordinating with FGF-induced angiogenesis [21,22,23,41,42]. Endothelial nitric oxide synthase (eNOS) is critical for ischemic remodeling and collateral arterial adaptation, leading to flow recovery in the hind limb ischemia (HLI) mouse model [24,43]. Recent research indicates that aspartate regulates endothelial translation machinery for VEGFR2 and FGFR1 synthesis via mTORC1 [44]. Long-term administration of L-arginine inhibits atherosclerosis and myointimal hyperplasia while enhancing angiogenesis in preclinical studies [45,46,47]. Additionally, short-term L-arginine supplementation in patients with cardiovascular risk factors has been shown to increase NO synthesis and improve vasoreactivity [35]. Consistent with these studies, the administration of LOLA increased the expression of VEGF and promoted angiogenesis in our CLI model. However, long-term administration of L-arginine in patients with PAD does not increase NO synthesis or improve vascular reactivity and may even pose a risk of harm [36]. Unlike administering arginine directly, LOLA induces an increase in arginine by adding reactants in the urea cycle, which may result in greater physiological efficacy and safety. Therefore, further long-term study with LOLA in the CLI model is necessary.

We believe it is challenging to uniformly apply methods and results from a specific mouse or rat model to other mouse or rat HLI models due to different perfusion recovery patterns dependent on the rate of arterial occlusion in mice and rats, as well as varying tissue necrosis in mouse strains [1,48,49,50]. Therefore, the HLI model and protocol in this study were designed to align with our hypotheses and aims. Unilateral proximal femoral artery ligation, without total excision or coagulation of its branches, allowed sufficient reperfusion between PODs 7 and 14, making it suitable for evaluating angiogenesis in the calf muscle [1]. Perfusion imaging was conducted solely on the ischemic hind limb without comparison to the non-ischemic contralateral limb. This approach aligns with the need for quantitative perfusion imaging in the affected lower extremity of CLI patients before and after revascularization [51]. Immediate post-operative perfusion in our HLI model experienced a 40% reduction compared to normal pre-operative levels. Subsequently, perfusion increased over 14 days, reaching a plateau between weeks three and four at approximately 60% recovery without any treatment, resembling the recovery pattern seen in a previous mouse HLI model [52]. While the 60% perfusion recovery plateau does not fully meet the diagnostic criteria of CLI in humans, it falls within at least a moderate degree of PAD [51,53]. Hepa-Merz can be administered to patients with hepatic encephalopathy, usually up to four ampoules daily (5 g per ampoule). If the patient is in a pre-coma state, the dose can be increased up to eight ampoules/24 h with a maximum infusion rate of 5 g/h, depending on the severity of the condition. This means that 308–615 mg/kg of Hepa-Merz can be administered per day for a 65 kg adult. Unfortunately, there is no standardized dosage of Herpa-Merz for administration to rat CLI models. Thus, the LOLA dosage was determined based on previous toxicity reports in Sprague Dawley rats (lethal dose 50%: 4.7 g/kg, no systemic toxicity between 1 and 4 g/kg) and the usual dosage for human application [27,54,55].

In the clinical setting, physicians encounter challenges in selecting an optimal treatment for patients with CLI due to a lack of high-level evidence to guide clinical decision-making [17,56,57,58,59,60]. Currently, endovascular revascularization is preferred for CLI due to lower morbidity and mortality compared to open surgery. However, the greater need for reinterventions in endovascular procedures results in a loss of cost-effectiveness [8,61,62]. Despite the increased use of endovascular interventions, overall amputation rates have not significantly improved [9,63]. Among promising gene therapies, hepatocyte growth factor—despite being the closest to human application—faced setbacks, as seen in the terminated phase III AnGes trial (ClinicalTrials.gov: NCT02144610) [17,64,65]. Various cell therapies, including bone marrow-derived mononuclear cells and mesenchymal stromal cells, studied in CLI, have shown no clinical efficacy to date [66,67]. Human pluripotent stem cell-derived endothelial cells, a potential candidate for cell therapy in CLI, have not yet entered clinical trials [68]. Hence, our study aimed to identify a drug for CLI treatment by leveraging a substance already in use for other human diseases, facilitating easier clinical application. A clinical trial with short-term and long-term administration of LOLA can be conducted, similar to previous studies on PAD with L-arginine. In addition, since LOLA is currently used for the clinical management of hepatic encephalopathy, we can assess the change in perfusion in the lower extremities of patients with hepatic encephalopathy and concurrent PAD after LOLA administration. The significantly lower cost of LOLA compared to endovascular interventional procedures or surgical bypass makes it an attractive option for clinical application, particularly given its established safety in managing hepatic encephalopathy. If further preclinical studies confirm LOLA’s feasibility in treating PAD based on the results of this study, clinical trials for LOLA administration will be actively pursued by investigators.

This study is subject to various limitations. Firstly, we exclusively examined the subacute phase of critical limb ischemia (CLI), and further investigation into the effects of LOLA on the chronic stage is warranted. Secondly, we did not include a robust mechanism of action study to couple with potential mechanisms for LOLA’s effects on angiogenesis and perfusion recovery, which is essential and should be pursued in subsequent research. We measured NO levels in samples from gastrocnemius muscles and plasma two days after the last LOLA injection. However, there was no significant difference between the three groups. Consequently, we speculate that L-arginine, L-citrulline, or NOS3 expression would yield similar results. We believe the interval between the last injection and sampling might have been too long. Therefore, we plan to conduct a future mechanistic study with a shorter interval, within 24 h, to evaluate the NO balance. Thirdly, we did not employ variable doses of LOLA, a consideration that will be essential for subsequent clinical trials. Fourthly, our study’s sample size was small, which may affect the power of the results. While our emphasis was on addressing the immediate requirements of CLI using a clinically available drug, future research should include preclinical investigations into the long-term administration of LOLA and clinical trials.

## 5. Conclusions

This study provides the first evidence of angiogenesis and improved blood flow during the subacute phase of hind limb ischemia using LOLA. Due to LOLA’s commercial availability, the application of LOLA treatment for PAD can be initiated more rapidly than other therapies that are still being developed.

## Data Availability

Data are contained within the article.
